# Improvement of ribonucleic acid production in *Cyberlindnera jadinii* and optimization of fermentation medium

**DOI:** 10.1186/s13568-024-01679-3

**Published:** 2024-02-15

**Authors:** Mengting Li, Shuhong Gao, Pengcheng Yang, Hejin Li

**Affiliations:** State Key Laboratory of Bioreactor Engineering, East China University of Science and Technology, Shanghai, 200237 China

**Keywords:** Atmospheric and room temperature plasma (ARTP), *Cyberlindnera jadinii*, Plackett–Burman, Ribonucleic acid (RNA)

## Abstract

To enhance the ribonucleic acid (RNA) productivity for industrial applications, this study employed strain screening and medium optimization to improve the content of RNA in *Cyberlindnera jadinii*. A rapid screening method, combining atmospheric and room temperature plasma mutagenesis, 48-deep-well plates fermentation, and microplate reader detection, was developed. A mutant strain named WB15 with high RNA content was successfully obtained, exhibiting the RNA content of 156 ± 4.5 mg/g DCW, 1.4 times of the starting strain CCTCC AY 92020. Furthermore, Plackett–Burman design and response surface methodology were employed to identify three significant factors (yeast extract, soybean peptone, and KH_2_PO_4_) affecting the RNA content. By utilizing the optimal medium composed of 13.43 g/L yeast extract, 12.12 g/L soybean peptone and 2.78 g/L KH_2_PO_4_, the RNA content of WB15 further increased to 184 ± 4.9 mg/g DCW. Additionally, the mutant strain WB15 exhibited a greater cellular width compared to AY 92020, along with increased growth rate and single-cell RNA content by 22% and 48.9%, respectively. Perturbations in ribosome assembly, specifically a reduction in the ratio of ribosomal proteins to ribosomal RNA of the large subunit, might indirectly contribute to the higher RNA content in the WB15 strain. Overall, the combination of rapid screening with fermentation medium optimization proved to be an effective approach for improving the RNA content of *C. jadinii*, thus facilitating the industrial production of RNA.

**Supplementary Information:**

The online version contains supplementary material available at 10.1186/s13568-024-01679-3.

## Introduction

Ribonucleic Acid (RNA) is a crucial macromolecule involved in gene expression and protein synthesis in organisms. Its hydrolysis products and derivatives have extensive applications as food additives and medical precursors. For instance, the disodium salts of 5′-inosine monophosphate and 5′-guanosine monophosphate can be utilized as food additives to enhance the flavor of food (Kurihara and Kashiwayanagi 2000). Furthermore, nucleotides, nucleosides and nucleobases obtained from the degradation of RNA serve as essential medical precursors, and their derivatives have demonstrated effectiveness in fighting against tumors and viruses (Ying et al. 2004).

In yeast, there are mainly three types of RNA participating protein biosynthesis: ribosomal RNA (rRNA), transfer RNA (tRNA), and messenger RNA (mRNA), which account for almost 80%, 15%, and 5% of the total RNA content, respectively (Rabl et al. 2011; Warner 1999). The primary role of rRNA is to form ribosomes with ribosomal proteins (RPs). In yeast cells, ribosomes are composed of a 40S small subunit and a 60S large subunit. The 40S small subunit is made up of 18S rRNA and 33 ribosomal proteins, while the 60S large subunit consist of 25S rRNA, 5.8S rRNA, 5S rRNA, and 46 ribosomal proteins (Rabl et al. 2011; Sergey 2012). The RNA content of yeast is closely associated with the levels of rRNA.

Currently, *Saccharomyces cerevisiae* and *Candida* species are widely used for the RNA production, because of their higher RNA content (Li et al. 2019; Khatun et al.^2013^). *Candida tropicalis* has been employed for RNA production and can achieve a maximum yield of 208 mg/g DCW (Yue et al. 2019). *S. cerevisiae* can yield up to 192.7 mg/g DCW (Guo et al. 2021). The RNA content of *Candida utilis* can reach up to 168 mg/g DCW (Jiang et al. 2017a). Although the previous studies have demonstrated the ability to achieve higher RNA content, it is worth noting that the production strains utilized are either not generally recognized as safe (GRAS) or unable to achieve high cell densities, rendering them unsuitable for industrial-scale production. *C. utilis* has been recognized as GRAS microorganism (Buerth et al. 2011) by the United States Food and Drug Administration (FDA), which can be applied to food production (Bekatorou et al. 2006; Boze et al. 1992). *C. utilis* has now been renamed to *Cyberlindnera jadinii* due to the high similarity in their genome sequences, and *C. utilis* is considered to be the anamorph state of *C. jadinii* (Kurtzman et al. 1979; Rupp et al. 2015; Sousa-Silva et al. 2021). *C. jadinii* possesses several advantageous characteristics, such as tolerance to broad temperature range (19–37 ℃) (Riley et al. 2016), Crabtree-negative effect (Schuler et al. 2012) and utilization of inexpensive substrate including molasses, inorganic nitrate sources, and even industrial wastewater (Buerth et al. 2016; Minoru and Hiroshi 2014). This means culture with *C. jadinii* can achieve higher cell density at robust culture condition such as ample oxygen supply with no ethanol production, broad temperature and varied inexpensive substrate for industrial RNA production. In the industrial production sector of RNA, it is important to have an excellent strain with higher RNA content to achieve higher cell density at robust culture condition.

High RNA-producing yeasts can be achieved through mutation. The commonly used physical mutagenesis method including ultraviolet mutagenesis is simple but with limited mutation rate. The recently developed atmospheric room temperature plasma (ARTP) mutagenesis is a novel mutagenesis strategy with highly active particles to cause immense damages at the genomes (Chen et al. 2010; Krishna et al. 2007; Huang et al. 2021; Zhang et al. 2014), which has been successfully applied to bacteria, fungi and microalgae, etc. (Liu et al. 2013; Qi et al. 2014; Wang et al. 2010). Combination of suitable high-throughput screening technology such as ultraviolet or visible light spectrophotometry with random mutagenesis can achieve desired strains rapidly and effectively (Xiong et al. 2022; Yu et al. 2022).

In this study, ARTP mutagenesis and microplate culture were applied to screen strains with higher RNA content based on the ultraviolet light absorbance of RNA at 260 nm. Additionally, the fermentation medium of the mutant was optimized for RNA production. The morphology, growth performance, single-cell RNA content, transcription levels of ribosomal genes were also investigated in the mutant strain WB15 to investigate the underlying factors contributing to the improved RNA production.

## Materials and methods

### Materials

Yeast extract and soybean peptone were purchased from Oxoid (Thermo Fisher Scientific, the United States of America). Glucose and corn steep liquor were industrial grade reagents stored at our laboratory. All other chemicals were purchased from Titan Scientific Co., Ltd. (Shanghai, China). 48-deep-well plates were purchased from Labgic Technology Co., Ltd. (Beijing, China).

### Strain, medium and culture conditions

The parent strain *C. jadinii* CCTCC AY 92020 used in this study, which was purchased from China Center for Type Culture Collection (CCTCC).

Solid medium (g/L): glucose 20, yeast extract 10, tryptone 20, agar 15.

Seed medium (g/L): glucose 40, corn steep liquor 15, KH_2_PO_4_ 2.34, MgSO_4_ 1.2, pH 6.5.

Fermentation medium (g/L): sucrose 50, yeast extract 10, soybean peptone 10, KH_2_PO_4_ 2.34, MgSO_4_ 1.2, FeSO_4_ 0.01, ZnSO_4_ 0.01, pH 5.5.

Cultivation conditions: a single colony was inoculated into either 48-deep-well plates or a 250 mL flask with 800 μL or 25 mL seed medium. Cultures were conducted at 30 ℃ for 18 h with a rotation speed of 220 rpm. The 48-deep-well plates culture were directly used for RNA analysis, while the flask fermentation was conducted with a 4% (v/v) inoculum in a 250 mL baffle flask containing 50 mL of fermentation medium. The culture was incubated at 30 ℃ for 8 h with a rotation speed of 220 rpm.

### ARTP mutation

*Cyberlindnera jadinii* AY 92020 was treated with ARTP mutagenesis breeding machine (ARTP-IIS, Si Qing Yuan Biotechnology Co., Ltd., Wuxi, China) equipped with a plasma generator, helium gas source, and regulator system. Yeast cells were cultured overnight, adjusted to the concentration of 10^7^ CFU/mL, then exposed to ARTP mutagenesis as follows: the radio frequency powered at 120 W, helium flow rate was maintained at 10 SLM, and the distance between the sample and nozzle was set to 2 mm (Li et al. 2008; Ottenheim et al. 2018). The mutagenized suspension was serially diluted, cultured at 30 ℃ for 2 days. The lethality rates were calculated as follows:

$${\text{Lethality}}(\% ) = ({{\text{T}}_0} - {{\text{T}}_1})/{{\text{T}}_0} \times 100$$

In which, T_0_ is the cell number without mutation, while T_1_ is the cell number after mutation treatment of different time.

### Dry cell weigh (DCW) measurement

10 mL of fermentation broth was centrifuged, washed twice, and dried at 80 ℃ until a constant weight. Additionally, a regression equation was established correlating the absorbance at 600 nm (OD_600_) with DCW as 0.4194 * OD_600_ + 1.0518.

### Extraction and measurement of RNA content

The RNA content was measured via perchloric acid extraction method with some modifications (Chuwattanakul et al. 2011). Following cultivation, the fermentation broth was centrifuged at 4000 rpm for 10 min to collect the cell pellets. After washing with 0.9% NaCl twice, the pellets were resuspended in 0.25 mol/L perchloric acid at 4 ℃ for 15 min, then centrifuged at 4000 rpm for 10 min to collect the pellets, resuspended in 0.5 mol/L perchloric acid at 75 ℃ for 15 min with gentle agitation. After a final centrifugation step, the supernatant was quantified at 260 nm using a microplate reader or ultraviolet–visible spectrophotometer after proper dilution.

The RNA content was calculated using the following equation:

$${\text{RNA content}}\,({\text{mg}}/{\text{gDCW}}) = ({\text{O}}{{\text{D}}_{260}} \times {\text{D}} \times 0.03365 \times {{\text{V}}_1})/({\text{DCW}} \times {{\text{V}}_2})$$

In which, OD_260_ is the absorbance of extracted supernatant at 260 nm, D is the dilution ratio; V_1_ is the volume of 0.5 mol/L perchloric acid solution, mL; V_2_ is the volume of fermentation broth, mL; 0.03365 corresponds to the RNA content in the solution to be tested when the absorbance is 1.0.

### Genetic stability of the mutant strain

The mutant strain exhibiting high RNA content was sequentially subcultured up to the 10th generation on agar slant cultures. Subsequently, the mutant strains were cultivated in shaking flasks to measure the RNA content and assess their genetic stability.

### Plackett–Burman and central composite designs

Plackett–Burman design is a valuable tool to identify significant factors using less experiments to screen multiple factors simultaneously. In the preliminary experiments, eight factors, including sucrose, yeast extract, soybean peptone, (NH_4_)_2_SO_4_, KH_2_PO_4_, MgSO_4_, FeSO_4_, and ZnSO_4_, had impact on the RNA content of *C. jadinii*. Using Design-Expert 8.0.6 software, a total of 12 experiments were designed as Table 1.

**Table 1:** Plackett–Burman design and experimental results

Run	Factors											RNA content/ (mg/g DCW)
	A	B	C	D	E	F	G	H	J	K	L	
1	1	1	− 1	1	1	1	− 1	− 1	− 1	1	− 1	159 ± 4.7
2	− 1	1	1	− 1	1	1	1	− 1	− 1	− 1	1	167 ± 6.2
3	1	− 1	1	1	− 1	1	1	1	− 1	− 1	− 1	168 ± 3.5
4	− 1	1	− 1	1	1	− 1	1	1	1	− 1	− 1	159 ± 7.0
5	− 1	− 1	1	− 1	1	1	− 1	1	1	1	− 1	167 ± 5.6
6	− 1	− 1	− 1	1	− 1	1	1	− 1	1	1	1	164 ± 7.2
7	1	− 1	− 1	− 1	1	− 1	1	1	− 1	1	1	161 ± 3.9
8	1	1	− 1	− 1	− 1	1	− 1	1	1	− 1	1	158 ± 5.8
9	1	1	1	− 1	− 1	− 1	1	− 1	1	1	− 1	160 ± 5.8
10	− 1	1	1	1	− 1	− 1	− 1	1	− 1	1	1	159 ± 4.3
11	1	− 1	1	1	1	− 1	− 1	− 1	1	− 1	1	163 ± 6.7
12	− 1	− 1	− 1	− 1	− 1	− 1	− 1	− 1	− 1	− 1	− 1	158 ± 4.0

A, sucrose; B, yeast extract; C, soybean peptone; D, (NH_4_)_2_SO_4_; F, KH_2_PO_4_; G, MgSO_4_; J, FeSO_4_; K, ZnSO_4_; E, H and L, dummy factors

Based on the results of Plackett–Burman design, the optimal concentration of significant factors was further examined using the path of steepest ascent. A central composite design with three significant factors was designed to optimize the concentrations of culture medium components.

### Scanning electron microscopy (SEM) analysis

The yeast cells collected at the 8-h of the fermentation process were separated by centrifugation and then washed twice with 0.1 mol/L phosphate buffer (pH 7.0). The cells were fixed with 2.5% glutaraldehyde overnight at 4 ℃. Subsequently, the cells were dehydrated using a series of ethanol solutions with increasing concentrations (50%, 70%, 80%, 90%, 95%, and 100%, v/v). Finally, the samples were dried by freeze dryer (FD5-3, GOLD SIM International Co., Ltd., Beijing, China), coated with a layer of gold spray, and observed by scanning electron microscope (Hitachi S3400-N, Hitachi, Tokyo, Japan), which was maintained at approximately 15 kV. The width and length of yeast cells were measured as follows: selecting cells with clear boundaries in the electron microscope images, utilizing a ruler or scale bar, clicking, and dragging the line tool on the image to align it precisely with the cell's boundary, and documenting the measured values.

### Quantitative real‑time PCR

The yeast cells were cultivated in fermentation medium and collected after centrifugation for RNA extraction. RNA extraction was performed using UNlQ-10 Column Trizol total RNA isolation kit (Sangon Biotech, Shanghai, China). Reverse transcription was carried out using TransScript^®^ II first-strand cDNA synthesis SuperMix (TransGen Biotech, Beijing, China). Quantitative real-time polymerase chain reaction (qPCR) was conducted using SuperReal PreMix Plus (SYBR Green) (Tiangen Biotech, Beijing, China) and the CFX96 touch real-time PCR detection system (Bio-Rad, Shanghai, China). The primers utilized in this experiment were shown in the Additional file 1: Table S1. The primer pairs UBC6-F and UBC6-R, 18S-F and 18S-R, 25S-F and 25S-R, RPL13-F and RPL13-R, RPS6-F and RPS6-R were employed to amplify genes of *UBC6*, 18S rRNA, 25S rRNA, *RPL13* and *RPS6*. The qPCR conditions were as follows: 95 ℃ for 15 min, followed by 40 cycles at 95 ℃ for 10 s and 60 ℃ for 32 s. The transcriptional level of gene *UBC6* which encodes ubiquitin-conjugating enzyme was used as an internal control (Guo et al. 2020).

### Statistical analysis

Three parallel samples were set in each group during the experiment. The data was presented as averages standard deviation. Analysis of variance (ANOVA) was conducted using IBM SPSS Statistics 25 to determine significant differences between the samples (*P* < 0.05).

## Results

### ARTP mutation

Mutation time is a key parameter affecting the mutation efficiency. Generally, a higher mutation efficiency is associated with increased DNA damage and higher lethality rate (Nyabako et al. 2020; Zhang et al. 2015). In this study, as depicted in Additional file 1: Fig. S1, lethality rate of *C. jadinii* exhibited a time-dependent pattern and reached 94.12% after a treatment time of 30 s. Consequently, a treatment time of 30 s was selected for mutating *C. jadinii* AY 92020. Following mutation, several hundreds of colonies were selected to culture in 48-deep-well plates.

### Screening of strains with high RNA content

**Fig. 1:**
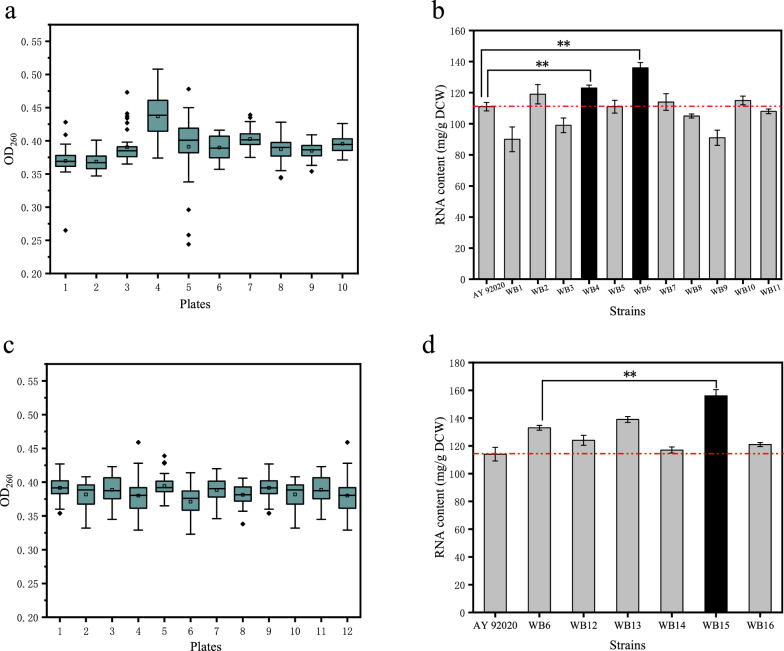
The screening results after ARTP.** a** The pre-screening results of 48-deep-well plates after the first round of ARTP. The mutants corresponding to the extreme outliers were selected for re-screening to determine its RNA content.** b** The results of re-screening after the first round of ARTP. ** means extreme significant (*P*< 0.01).** c** The pre-screening results of 48-deep-well plates after the second round of ARTP.** d** The results of re-screening after the second round of ARTP. ** means extreme significant (*P* < 0.01)

Two rounds of ARTP mutagenesis were performed for the screening with high RNA content. During the first round of screening in 48-deep-well plates culture, the mutants corresponding to the extreme outliers with higher OD_260_ values were selected for re-screening in flask culture to determine its RNA content. 11 mutants were selected from a total of 398 mutants (Fig. 1a) in the first round of screening, and then re-screened in flask (Fig. 1b). Among them, strain WB6 exhibited the highest RNA content (136 ± 3.4 mg/g DCW), which was 1.2 times that of AY 92020 (*P* < 0.01).

The second round of screening was the same as the first round of screening after another ARTP mutation with strain WB6 as parent strain. Five mutants from 478 mutants with high OD_260_ were screened out in 48-deep-well microplate culture (Fig. 1c). Then the 5 selected strains were re-screened in flask fermentation and the results were depicted in Fig. 1d. Among these strains, WB15 exhibited the most significant increase in RNA content, reaching 156 ± 4.5mg/g DCW, which was1.2 times of the second round starting strain WB6 (*P* < 0.01) and 1.4 times of the parent strain AY 92020 (*P* < 0.01).

### Genetic stability of WB15

After undergoing mutation, the resulting mutants may experience revert mutation, making it necessary to cultivate them for several generations to determine their genetic stability. In this study, the mutant strain WB15 was continuously passaged 10 times on slant culture, and the RNA content of these mutants remained consistent, ranging between 156-162mg/g DCW across all ten generations (Additional file 1: Fig. S2). Analysis using one-way ANOVA revealed no significant difference in RNA content between the ten generations, suggesting that the mutant strain WB15 exhibited genetic stability.

### Plackett–Burman design

Plackett–Burman design was utilized to identify the factors that had a significant impact on the RNA content of strain WB15. Eight factors, including sucrose, yeast extract, soybean peptone, (NH_4_)_2_SO_4_, KH_2_PO_4_, MgSO_4_, FeSO_4_, and ZnSO_4_ were selected according to their impact on the RNA content of *C. jadinii*. A total of 11 factors were tested in 12 experimental runs, and the resulting RNA contents of WB15 were presented in Table 1. The coded levels and actual values of each factor were presented in Additional file 1: Table S2. The pareto chart (Additional file 1: Fig. S3) illustrated the effect of the main factors on the RNA content. Table 2 presented the estimated effect of each factor on the RNA content. The ANOVA revealed that four factors, namely soybean peptone, yeast extract, KH_2_PO_4_ and MgSO_4_, had a statistically significant impact on the RNA content (*P* < 0.05). Among these factors, the top three in terms of their influence on the RNA content was soybean peptone, yeast extract and KH_2_PO_4_. So, these three factors were selected for further optimization.

**Table 2:** Analysis of variable for Plackett–Burman design

Source	Sum of	df	Mean	F	p-value	Rank	
	Squares		Square	Value	Prob > F		
Model	145	4	36.25	25.59	0.0003		Significant
B-yeast extract	30.08	1	30.08	21.24	0.0025	3	
C-soybean peptone	52.08	1	52.08	36.76	0.0005	1	
F-KH_2_PO_4_	44.08	1	44.08	31.12	0.0008	2	
G-MgSO_4_	18.75	1	18.75	13.24	0.0083		
Residual	9.92	7	1.42				
Cor Total	154.92	11					

R-Squared, 0.9360; Adj R-Squared,  0.8994; Adeq Precisior,  14.534

### The path of steepest ascent

The optimal concentration of soybean peptone, yeast extract and KH_2_PO_4_ was further examined using the path of steepest ascent. The results were presented in Table 3 and the RNA content exhibited a continuous increase from trial number 1 to 3, followed by a decrease from trial 4 onwards. Consequently, the conditions corresponding to trial number 3, which included yeast extract 13.4 g/L, soybean peptone 12.2 g/L and KH_2_PO_4_ 2.78 g/L, were selected for subsequent central composite design.

**Table 3:** The experimental design of the steepest ascent

Trial No	Yeast extract	Soybean peptone	KH_2_PO_4_	RNA content/ (mg/g DCW)
1	15	10	2.34	169 ± 1.9
2	14.2	11.1	2.56	171 ± 9.4
3	13.4	12.2	2.78	177 ± 4.2
4	12.6	13.3	3	171 ± 1.2
5	11.8	14.4	3.22	171 ± 2.8
6	11	15.5	3.44	169 ± 2.3
7	10.2	16.6	3.66	168 ± 2.4
8	9.4	17.7	3.88	167 ± 6.0

CCD, Central composite design

According to the results of the steepest ascent design, a three-factor and five-level experiment was designed using CCD (Additional file 1: Table S3). The complete experimental design matrix, consisting of 20 runs and their corresponding RNA content, was presented in Table 4. To assess the adequacy and significance of the second-order polynomial model, ANOVA was conducted, and the results are shown in Additional file 1: Table S4.

**Table 4:** Central composite design and experimental results

Run	Point type	A- yeast extract	B- soybean peptone	C- KH_2_PO_4_	RNA content/ (mg/g DCW)
1	Factorial	12.60	11.10	2.56	175 ± 5.7
2	Factorial	14.20	11.10	2.56	172 ± 6.5
3	Factorial	12.60	13.30	2.56	164 ± 2.9
4	Factorial	14.20	13.30	2.56	171 ± 1.2
5	Factorial	12.60	11.10	3.00	171 ± 3.9
6	Factorial	14.20	11.10	3.00	168 ± 3.9
7	Factorial	12.60	13.30	3.00	168 ± 1.7
8	Factorial	14.20	13.30	3.00	173 ± 3.4
9	Axial	12.05	12.20	2.78	164 ± 2.5
10	Axial	14.75	12.20	2.78	166 ± 2.9
11	Axial	13.40	10.35	2.78	166 ± 3.5
12	Axial	13.40	14.05	2.78	164 ± 1.2
13	Axial	13.40	12.20	2.41	172 ± 6.5
14	Axial	13.40	12.20	3.15	175 ± 3.5
15	Center	13.40	12.20	2.78	183 ± 4.8
16	Center	13.40	12.20	2.78	186 ± 6.4
17	Center	13.40	12.20	2.78	180 ± 5.7
18	Center	13.40	12.20	2.78	184 ± 0.8
19	Center	13.40	12.20	2.78	187 ± 6.0
20	Center	13.40	12.20	2.78	184 ± 2.6

The terms 'center', 'axial' and 'factorial' correspond to distinct point types employed in the experimental design

Regression analysis was carried out by Design-Expert 8.0.6 to derive the second-order polynomial equation:

$${\text{RNA content}}\, = \,{183}.{89}\, + \,0.{69}*{\text{A}} - 0.{98}*{\text{B}}\, + \,0.{22}*{\text{C}}\, + \,{2}.{25}*{\text{A}}*{\text{B}} - 0.{25}*{\text{A}}*{\text{C}}\, + \,{1}.{75}*{\text{B}}*{\text{C}} - {6}*{\text{A}}^{{2}} - {6}*{\text{ B}}^{{2}} - {3}*{\text{C}}^{{2}}$$

ANOVA revealed that the regression model was highly significant, F-value (16.45, *P* < 0.0001). The accuracy of this regression model was further confirmed by an insignificant lack of fit value (*P* = 0.3495). Moreover, the *P* values of AB, A^2^, B^2^ and C^2^ were all less than 0.05, indicating that these factors were significant in the model. The correlation coefficient R-Squared of the regression equation was 0.9367, while Adj R^2^ (adjusted R-squared, 0.8798) was consistent with Pred R^2^ (predicted R-squared, 0.6799). These values suggested that the regression equation was reliable. The adequacy precision, represented by the signal-to-noise ratio of the model, was calculated to be 11.048, which was greater than 4. This indicated a high level of reliability for the model.

### Verification of the flask fermentation results

The software predicted that the maximum RNA content would be 184 mg/g DCW when the medium formula was yeast extract 13.43 g/L, soybean peptone 12.12 g/L and KH_2_PO_4_ 2.78 g/L. To validate the improvement achieved through optimization, the RNA content of strain WB15 was measured after flask fermentation. The maximum RNA content reached 184 ± 4.9 mg/g DCW, which was very close to the predicted value. This indicated that the model is highly feasible. Furthermore, the RNA content of 184 ± 4.9 mg/g DCW was 1.2 times (*P* < 0.01) of the control medium (153 ± 3.5 mg/g DCW), demonstrating a significant improvement.

### Morphological observation of the parent strain and the mutant strain

**Fig. 2:**
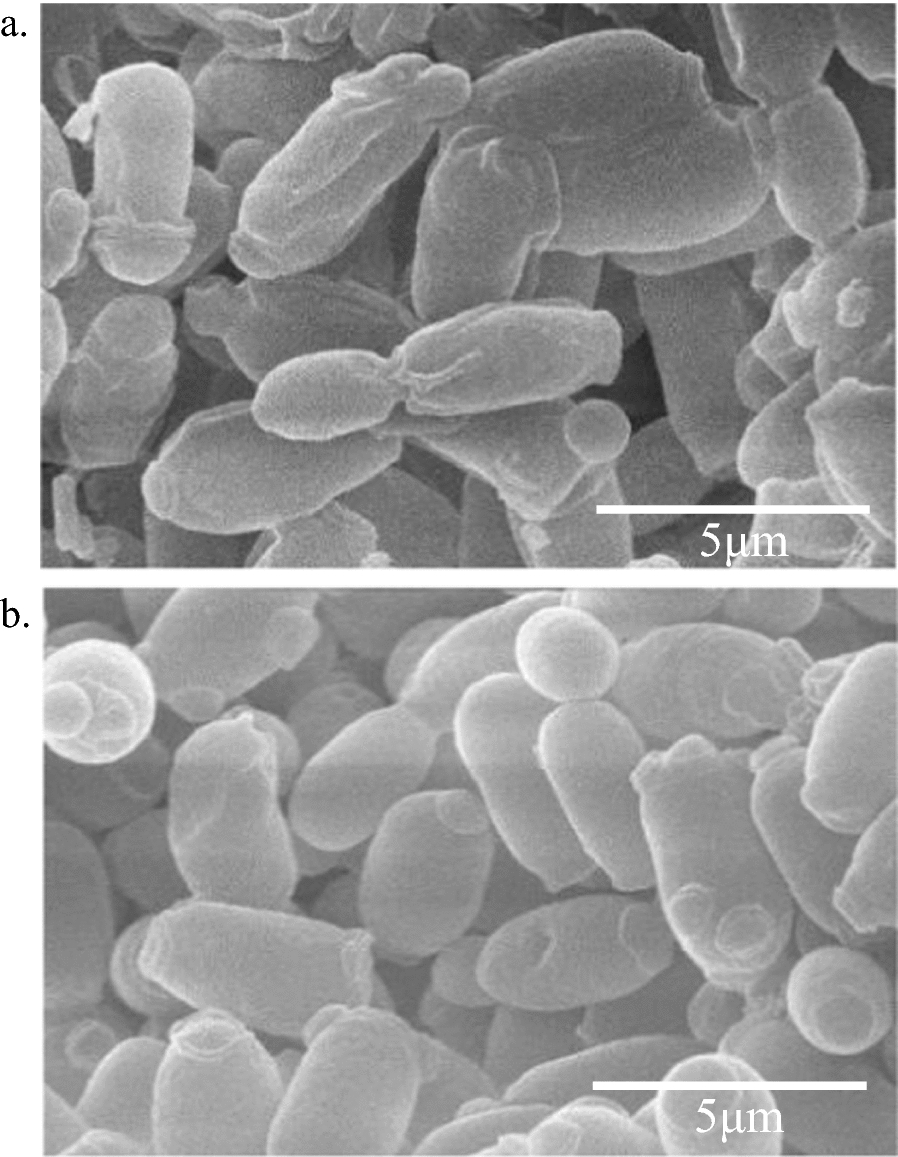
The SEM images of AY 92020 and WB15 (magnification, × 8000).** a** AY 92020,** b** WB15

The cell size and morphology of the parent strain AY 92020 and the mutant strain WB15 was analyzed using SEM (Fig. 2). In Fig. 2, it can be observed that the cells of WB15 were relatively plump with a smooth and intact surface. Clear bud marks resulting from cell proliferation on the yeast surface were visible, and there was minimal cell adhesion. In contrast, defects and shriveled cells were observed in the AY 92020 cells, with increased cell adhesion, a rough surface, and numerous folds. Table 5 showed that there was no significant difference in cell length, but the width of WB15 was 2.14 ± 0.20 μm, which was almost 10% wider than AY 92020 (1.96 ± 0.19 μm) (*P* < 0.01). It had been shown that *S. cerevisiae* with a high growth rate had a much higher RNA content (Kief and Warner 1981) and the cell size was significantly larger than the size of the parent strain when constructed with overexpressing *FHL1*, *IFHL1*, and *SSF2* and deleting *HRP1*, possibly due to the accumulation of more RNA in the cell (Guo et al. 2020).

**Table 5:** The cell length and width for AY 92020 and WB15 (*n* = 20)

Strains	Length(μm)	Width(μm)
AY 92020	4.04 ± 0.59	1.96 ± 0.19
WB15	4.03 ± 0.42	2.14 ± 0.2^**^

The width and length of twenty cells from strains AY 92020 and WB15 were measured in the electron microscope images

^**^Means extreme significant (*P* < 0.01)

### Comparison of fermentation parameters

**Fig. 3:**
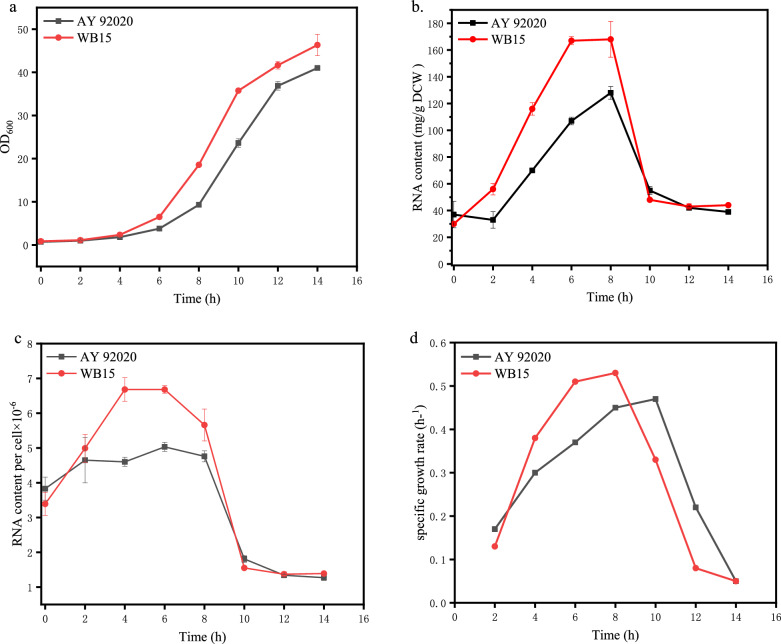
Comparison of fermentation parameters between the parent strain AY 92020 and the mutant strain WB15.** a** OD_600_,** b** RNA content,** c** single-cell RNA content,** d** specific growth rate.

The growth curves and RNA content of the parent strain AY 92020 and the mutant strain WB15 were measured as showed in Fig. 3a and b. The single-cell RNA content and specific growth rate were also calculated and displayed in Fig. 3c and d.

It was shown that the mutant strain WB15 exhibited faster growth compared to the parent strain AY 92020, with a final OD_600_ reaching 46.3, which was 1.1 times of the parent strain AY 92020. The RNA content in WB15 reached to 168 mg/g DCW at 8 h, while the parent strain had a maximum RNA content of 128 mg/g DCW at 8 h. The maximum specific growth rate of WB15 (0.44 h^−1^) was 22% higher than that of the parent strain (0.36 h^−1^). In Fig. 3c, the RNA content in single cell of AY 92020 remained at approximately 4.5 × 10^–6^ mg RNA/cell at 2–8 h, whereas the single-cell RNA content of the mutant strain WB15 increased continuously at 2–6 h and reached the maximum value 6.7 × 10^–6^ mg RNA/cell, 48.9% higher than that of AY 92020. The above results indicated that the mutant strain had a higher growth rate, and the single-cell RNA content could increase with the increasement of specific growth rate during the logarithmic growth phase. The findings were consistent with previous studies in which yeast cells with a high growth rate had a much higher RNA content (Waldron and Lacroute 1975). The parent strain AY 92020 also exhibited an increase in single-cell RNA content with the increase in specific growth rate, but it ceased to increase after reaching to 5.03 × 10^–6^ mg RNA/cell. This suggested that the mutant strain WB15 had surpassed some nutritional or growth restriction to achieve a higher single-cell RNA content.

### Transcription levels of rRNA and RPs

**Fig. 4:**
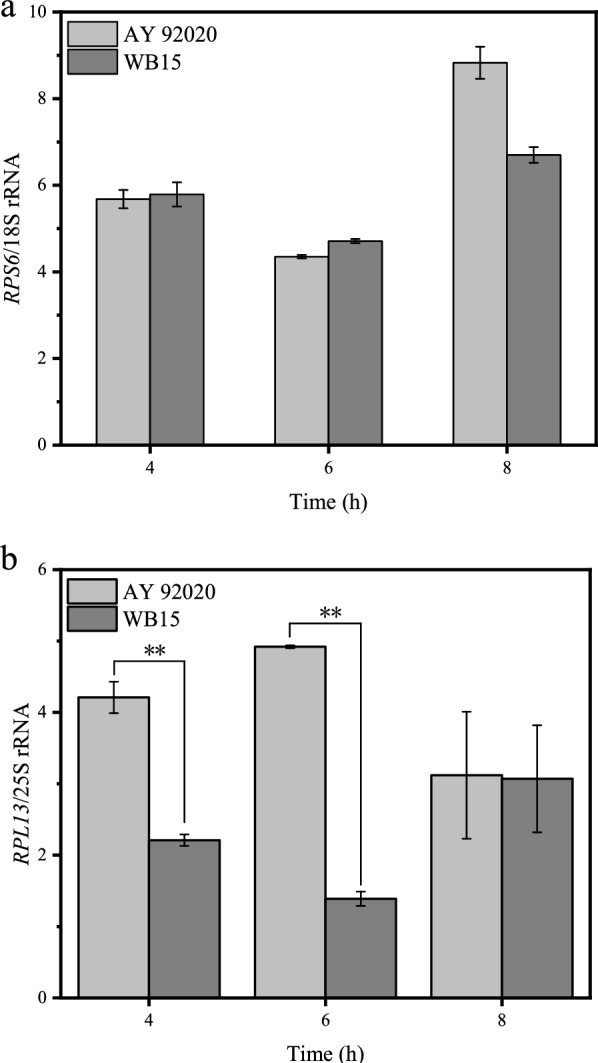
Relative transcription level of AY 92020 and WB15.** a*** RPS6*/18S rRNA,** b*** RPL13*/25S rRNA. The values were measured at 4 h, 6 h and 8 h of fermentation

In rapidly growing yeast cells, approximately 80% of total cellular RNA is composed of rRNA, and nearly 50% of all RNA polymerase II transcription initiation events occur on RPs (Lempiaeinen and Shore 2010). The synthesis of ribosome requires the coordinated expression of multiple steps, particularly the transcription of rDNA and RPs. Various rRNAs and RPs are produced in equimolar amounts, and their synthesis is tightly regulated by various growth conditions (Fromont-Racine et al. 2003). The RPs-to-rRNA ratio of eukaryotic small subunit is almost 1:1 (Wilson and Doudna Cate 2012). To investigate the reason for the higher single-cell RNA content in the mutant strain WB15, the transcription level of genes encoding large and small subunits were measured using qPCR. We chose genes of 25S rRNA, 18S rRNA, *RPL13* and *RPS6* to represent the transcription of rDNA and RPs of large subunit genes and small subunit genes, respectively. As shown in Fig. 4, the ratio of the values of *RPS6* to 18S rRNA and *RPL13* to 25S rRNA were compared between AY 92020 and WB15. The ratio of *RPS6* to 18S rRNA was of no significant difference during the fermentation process, while the ratio of *RPL13* to 25S rRNA were 4.2-fold and 2.2-fold at 4 h, 4.9-fold and 1.4-fold at 6 h for strain WB15 and AY 92020, respectively. There was no significant difference in the assembly ratio of RPs to rRNA in small subunit between AY 92020 and WB15, but the assembly of large subunit required less RPs in WB15. These results might indicate that the mutant strain WB15 required less precursor and energy to synthesize RPs, which resulted in the increasement of rRNA synthesis. Consequently, the decline in the RPs-to-rRNA ratio of large subunit might indirectly contribute to the higher RNA content of WB15.

## Discussion

Microorganisms serve as the primary source for industrial-scale RNA production, making the development of high RNA content strains crucial for this process. ARTP mutagenesis is a novel strategy for inducing DNA damage in cells, altering metabolic activities and genetic characteristics. ARTP mutagenesis has been successfully used many kinds of strain such as *Zygosaccharomyces rouxii* for improving the quality of soy sauce (Guo et al. 2019), *Streptomyces fradiae* for neomycin production (Yu et al.^2022^) and *S. mobaraensis* for transglutaminases production (Jiang et al. 2017b). In this study, we developed a rapid method using 48-deep-well microplate fermentation and fast screening according the absorbance of RNA at 260 nm to obtain *C. jadinii* mutants with high RNA content. A mutant strain named WB15 was obtained, in which the RNA content was 1.4 times of the parent strain AY 92020 (156 ± 4.5 mg/g DCW).

The composition and concentration of medium affected the growth and metabolism of yeast (Guo et al. 2022). Through medium optimization using Plackett–Burman design, we identified that soybean peptone, yeast extract, and KH_2_PO_4_ significantly impacted the RNA content of the WB15 strain. The path of steepest ascent and CCD were applied to further optimize the concentrations of soybean peptone, yeast extract and KH_2_PO_4_. With these optimized conditions, the RNA content reached 184 ± 4.9 mg/g DCW, which was 1.2 times of the control medium. Yeast extract and soybean peptone, rich in amino acids and other nitrogen sources, might enhance RNA synthesis capacity of *C. jadinii* by providing a greater supply of nutrients for growth. The addition of KH_2_PO_4_ might regulate the pondus hydrogenii of the culture medium and provide phosphorus for RNA synthesis, thereby increasing the synthesis of ribonucleotides and ultimately boosting RNA content.

The highest reported RNA content of 208 mg/g DCW was obtained in *C. tropicalis*, which exhibited the DCW of approximately 1.0 g/L and the RNA production of approximately 0.21 g/L (Yue et al. 2019). Diethyl sulfate was conducted to facilitate breeding in *S. cerevisiae* with an RNA content of 192.7 mg/g DCW (Guo et al. 2021). However, the yield was not high, with OD_600_ below 1, and the RNA production was less than 0.3 g/L. The RNA content of *C. jadinii* WB15 (156 ± 4.5 mg/g DCW) was lower than that of *S. cerevisiae* BY23-195 as mentioned above, but the OD_600_ of WB15 was 18.6 (DCW 8.54 g/L), and the RNA production was 1.33 g/L. The relatedly high RNA content and cell yield of *C. jadinii* WB15 give it a competitive advantage in industrial RNA production.

Ribosomes, as the central components of protein synthesis machinery, play a crucial role in determining the rate of protein synthesis and consequently influence cell growth and division (Warner 1999; Goodfellow and Zomerdijk 2013; von der Haar 2008). The synthesis of rRNA needs to be tightly regulated to ensure that the levels of mature rRNA and ribosomal protein RPs-related genes are in conjunction with the growth demands of the cell (Lempiaeinen and Shore 2010; Shore et al. 2021). In this study, the transcription level of *RPS6*/18S rRNA and *RPL13*/25S rRNA between AY 92020 and WB15 were measured. The ratio of *RPS6* to 18S rRNA showed no significant difference. However, the ratio of *RPL13* to 25S rRNA were 4.2-fold and 2.2-fold at 4 h, 4.9-fold and 1.4-fold at 6 h for strain WB15 and AY 92020, respectively. These results indicated that WB15 required fewer RPs for the assembly of the large subunit and allocated more energy to rRNA synthesis while reduced RPs synthesis, potentially contributing to the increase in RNA content.

We also measured the size of the mutant strain with SEM. The width of *C. jadinii* WB15 was 2.14 ± 0.20 μm, whereas AY 92020 exhibited a width of 1.96 ± 0.19 μm. Furthermore, the mutant strain WB15 exhibited increased growth rate and single-cell RNA content by 22% and 48.9% compared to AY 92020. The results showed that *C. jadinii* WB15 exhibited a larger cell width and higher growth rate compared to the parental strain AY92020. It had been shown that yeast cells with a high growth rate have a much higher RNA content (Kief and Warner 1981) and the cell size was significantly larger than the size of the parent strain when constructed with overexpressing *FHL1*, *IFHL1*, and *SSF2* and deleting *HRP1*, possibly due to the accumulation of more RNA in the cell (Guo et al. 2020). It had been showed that the rate of RNA polymerase I which participated in rRNA precursor synthesis in polyploid cells was increased in proportion to the cell size and genomic copy number (Pe´rez-Ortı´n et al. 2021). The reasons of an increased RNA content in *C. jadinii* WB15 might attributed to an increased transcription rate of RNA polymerase I, which need further exploration.

In conclusion, a mutant strain named *C. jadinii* WB15 was generated using combing ARTP mutagenesis and high-throughput screening method. Through medium optimization, RNA content of WB15 could reach 184 ± 4.9 mg/g DCW, which is the highest RNA content in *C. jadinii* reported so far. This study successfully demonstrated the effectiveness of combining ARTP mutagenesis and medium optimization can effectively improve RNA content of *C. jadinii*. Further investigations will involve genomic and transcriptomic sequencing of WB15 and AY 92020 strains to identify genomic variations and gene expression differences to underly molecular mechanisms associated with RNA content in *C. jadinii*.

### Supplementary Information

**Additional file 1:** **Figure S1.** The relationship between ARTP treating time and lethality. **Figure S2.** The genetic stability of WB15. **Figure S3.** Pareto chart of Plackett–Burman design. B: yeast extract; C: soybean peptone; F: KH_2_PO_4_; G: MgSO_4_. **Table S1.** Sequences of primers used in this study. **Table S2.** Factors and levels of Plackett-Burman design. **Table S3.** Factors and levels of central composite design. **Table S4.** ANOVA of variable for central composite design. R-Squared,  0.9367; Adj R-Squared,  0.8798; Adeq precisior,  11.048.

## Data Availability

All data have been included into the manuscript or the supplementary material.

## Abbreviations

ARTP: Atmospheric and room temperature plasma
RNA: Ribonucleic acid
DCW: Dry cell weight
CFU: Colony forming unit
SEM: Scanning electron microscopy
qPCR: Quantitative real-time polymerase chain reaction
ANOVA: Analysis of variance
CCD: Central composite design
