# Supplementary material for: Improvement of ribonucleic acid production in Cyberlindnera jadinii and optimization of fermentation medium
Source: AMB Express. 2024 Feb 15;14:24. doi: 10.1186/s13568-024-01679-3 (PMC10869677; doi:10.1186/s13568-024-01679-3)
Supplement: Supplementary file 1 — Additional file 1: Figure S1. The relationship between ARTP treating time and lethality. Figure S2. The genetic stability of WB15. Figure S3. Pareto chart of Plackett–Burman design. B: yeast extract; C: soybean peptone; F: KH2PO4; G: MgSO4. Table S1. Sequences of primers used in this study. Table S2. Factors and levels of Plackett-Burman design. Table S3. Factors and levels of central composite design. Table S4. ANOVA of variable for central composite design. R-Squared, 0.9367; Adj R-Squared, 0.8798; Adeq precisior, 11.048. [file 13568_2024_1679_MOESM1_ESM.docx]

**Supplementary Information**

Journal: AMB Express
Manuscript title: **Improvement of ribonucleic acid production in *Cyberlindnera jadinii* and optimization of fermentation medium**

Mengting Li^1^, Shuhong Gao^1*^, Pengcheng Yang^1^, Hejin Li^1^

*Corresponding author: Shuhong Gao, E-mail: [shhgao@ecust.edu.cn](mailto:shhgao@ecust.edu.cn)

^1^State Key Laboratory of Bioreactor Engineering, East China University of Science and Technology, Shanghai 200237, China

****Figure S1 The relationship between ARTP treating time and lethality

Figure S2 The genetic stability of WB15.


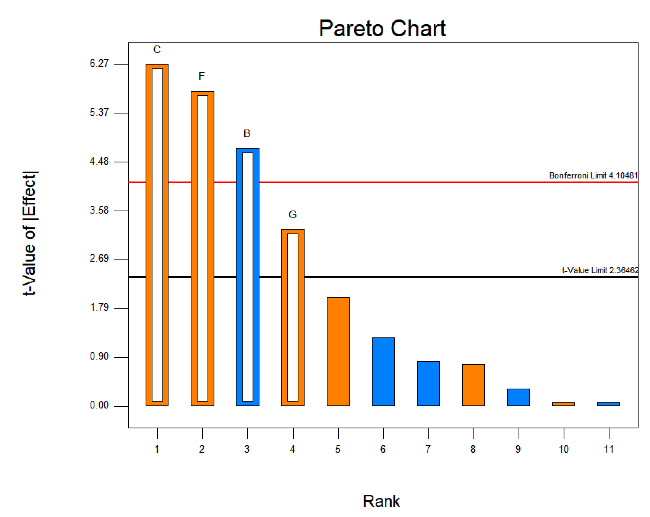
Figure S3 Pareto chart of Plackett-Burman design. B: yeast extract; C: soybean peptone; F:KH_2_PO_4_; G: MgSO_4_

**Table S1 Sequences of primers used in this study**

| Gene | Primers' name | 5'→ 3'DNA sequences |
| --- | --- | --- |
| *UBC6* | UBC6-F | ATCCTGATACATGGCAGCCG |
|  | UBC6-R | GGTTGGCTCATCACCAGTCA |
| 18S rRNA | 18S-F | AAACGGCTACCACATCCAAGG |
|  | 18S-R | CACCAGACTTGCCCTCCAAT |
| 25S rRNA | 25S-F | ATTGTCAGGTGGGGAGTTTGG |
|  | 25S-R | GGCCACACTTTCATGGTTTGTAT |
| *RPL13* | RPL13-F | TGACCAAGCCGGTAAGAAGG |
|  | RPL13-R | CAACTGGTCTAAGAGCGTCCA |
| *RPS6* | RPS6-F | CAACGGGTCCCAAAAGACCT |
|  | RPS6-R | CTTGTCGTTACCACCGCTGA |

**Table S2 Factors and levels of Plackett-Burman design**

| Variables(g/L) | Code | Low (-1) | High (+1) |
| --- | --- | --- | --- |
| Sucrose | A | 50 | 75 |
| Yeast extract | B | 10 | 15 |
| Soybean peptone | C | 10 | 15 |
| (NH_4_)_2_SO_4_ | D | 5 | 7.5 |
| KH_2_PO_4_ | F | 2.34 | 3.51 |
| MgSO_4_ | G | 1.2 | 1.8 |
| FeSO_4_ | J | 0.01 | 0.015 |
| ZnSO_4_ | K | 0.01 | 0.015 |

**Table S3** **Factors and levels of central composite design**

| Variables(g/L) | Code | Values | | | | |
| --- | --- | --- | --- | --- | --- | --- |
|  |  | -1.682 | -1 | 0 | 1 | 1.682 |
| Yeast extract | A | 12.05 | 12.6 | 13.4 | 14.2 | 14.75 |
| Soybean peptone | B | 10.35 | 11.1 | 12.2 | 13.3 | 14.05 |
| KH_2_PO_4_ | C | 2.41 | 2.56 | 2.78 | 3 | 3.15 |

**Table S4 ANOVA of** **variable for central composite design**

| Source | Sum of | df | Mean | F | *P*-value |  |
| --- | --- | --- | --- | --- | --- | --- |
|  | Squares |  | Square | Value | Prob > F |  |
| Model | 1083.36 | 9 | 120.37 | 16.45 | < 0.0001 | significant |
| A-yeast extract | 6.42 | 1 | 6.42 | 0.88 | 0.3710 |  |
| B-soybean peptone | 13.08 | 1 | 13.08 | 1.79 | 0.2109 |  |
| C-KH_2_PO_4_ | 0.68 | 1 | 0.68 | 0.09 | 0.7669 |  |
| AB | 40.50 | 1 | 40.50 | 5.53 | 0.0405 |  |
| AC | 0.50 | 1 | 0.50 | 0.07 | 0.7991 |  |
| BC | 24.50 | 1 | 24.50 | 3.35 | 0.0972 |  |
| A^2 | 518.91 | 1 | 518.91 | 70.90 | < 0.0001 |  |
| B^2 | 518.91 | 1 | 518.91 | 70.90 | < 0.0001 |  |
| C^2 | 129.30 | 1 | 129.30 | 17.67 | 0.0018 |  |
| Residual | 73.19 | 10 | 7.32 |  |  |  |
| Lack of Fit | 43.19 | 5 | 8.64 | 1.44 | 0.3495 | not significant |
| Pure Error | 30.00 | 5 | 6 |  |  |  |
| Cor Total | 1156.55 | 19 |  |  |  |  |

R-Squared=0.9367, Adj R-Squared=0.8798, Adeq precisior=11.048
